# Optimization of a Lower Irrigation Limit for Lettuce Based on Comprehensive Evaluation: A Field Experiment

**DOI:** 10.3390/plants13060853

**Published:** 2024-03-15

**Authors:** Maomao Hou, Houdong Zhang, Hiba Shaghaleh, Jingnan Chen, Fenglin Zhong, Yousef Alhaj Hamoud, Lin Zhu

**Affiliations:** 1 College of Horticulture, Fujian Agriculture and Forest University, Fuzhou 350000, China; 2 College of Agricultural Science and Engineering, Hohai University, Nanjing 210098, China; 3 Nanjing Institute of Environmental Sciences, Nanjing 210000, China

**Keywords:** lettuce, drip irrigation, emission reduction, entropy weight coefficient

## Abstract

When optimizing irrigation methods, much consideration is given to crop growth indicators while less attention has been paid to soil’s gaseous carbon (C) and nitrogen (N) emission indicators. Therefore, adopting an irrigation practice that can reduce emissions while maintaining crop yield and quality is of great interest. Thus, open-field experiments were conducted from September 2020 to January 2022 using a single-factor randomized block design with three replications. The lettuce plants (“*Feiqiao Lettuce No.1*”) were grown using four different irrigation methods established by setting the lower limit of drip irrigation to 75%, 65%, and 55% of soil water content at field capacity corresponding to DR1, DR2, and DR3, respectively. Furrow irrigation (FI) was used as a control. Crop growth indicators and soil gas emissions were observed. Results showed that the mean lettuce yield under DR1 (64,500 kg/ha) was the highest, and it was lower under DR3 and FI. The lettuces under DR3 showed greater concentrations of crude fiber, vitamin C, and soluble sugar, and a greater nitrate concentration. Compared with FI, the DR treatments were more conducive to improving the comprehensive quality of lettuce, including the measured appearance and nutritional quality. Among all the irrigation methods, FI had the maximum cracking rate of lettuce, reaching 25.3%, 24.6%, and 22.7%, respectively, for the three continuous seasons. The stem cracking rates under DR2 were the lowest—only 10.1%, 14.4%, and 8.2%, respectively, which were decreased to nearly half compared with FI. The entropy model detected that the weight coefficient evaluation value of DR2 was the greatest, reaching 0.93, indicating that the DR2 method has the optimal benefits under comprehensive consideration of water saving, yield increase, quality improvement, and emission reduction.

## 1. Introduction

Irrigation is the leading water resource consumer and a key factor affecting soil greenhouse gas emissions [1]. Irrigation can change the conditions of soil moisture, temperature, oxygen, and microorganisms, thus influencing the production and release of greenhouse gases such as carbon dioxide (CO_2_), methane (CH_4_), and nitrous oxide (N_2_O) [2,3]. Different irrigation approaches affect the soil’s carbon © and nitrogen (N) emissions differently [4]. Earlier studies have shown that water-saving irrigation techniques such as drip irrigation (DI) and micro sprinkler irrigation can reduce CH_4_ emissions but may increase N_2_O emissions; however, conventional irrigation techniques such as flood irrigation have shown opposite impacts [5,6]. In addition, the irrigation amount also markedly influences the C/N discharges [7,8]. However, lower C and N discharges are rarely considered when designing irrigation tactics.

Lettuce is an important vegetable crop. It is rich in soluble sugar, vitamin C (Vc), and mineral nutrients. Irrigation impacts the crop’s development indicators, such as leaf area index, plant height, and stem diameter, and quality indicators, such as fiber, nitrate, and soluble solids contents of lettuce [9]. Therefore, crop quality, yield, and water use efficiency should be considered when adopting an irrigation method for lettuce, similar to other vegetable crops. However, there is limited information on the stem cracking rate of lettuce. Stem cracking of lettuce is caused by excessive irrigation after high temperatures and long-term drought, resulting in the rapid growth of fleshy stems and thus bursting of the external xylem. Recently, stem cracking has been the main problem restricting the development of the lettuce industry in southern China [10]. Therefore, the cracking rate under different irrigation methods is important and should be considered when optimizing water-saving irrigation approaches. Thus, when adopting drip irrigation strategies, yield and quality indicators and environmental indicators such as greenhouse gas emissions should be considered [11].

Water-efficient irrigation strategies should achieve environmental sustainability while enhancing water use efficiency and conservation. The optimization of irrigation practice is a decision that involves multiple objectives and multiple indicators. Multiple objectives refer to multiple irrigation systems designed by the experiment, and multiple indicators refer to the indicators that need to be considered, such as crop yield, quality, and water use efficiency [12]. The optimization of irrigation systems often relies on the designer’s expertise, which leads to the reasonable and scientific adoption of an irrigation strategy. Therefore, several mathematical models, including the projection pursuit classification, the analytic hierarchy process, and the fuzzy comprehensive evaluation method, have been introduced to the decision-makers of agricultural plans. Thus, earlier research has used the entropy evaluation method to optimize agricultural water conservancy tactics. However, the application of the entropy method in the optimization of irrigation systems for lettuce has not been investigated yet. Therefore, the current study hypothesizes that the different irrigation strategies would impact lettuce yield, quality, and carbon and nitrogen emissions. To test the assumption mentioned above, the present study evaluated the effects of different designed irrigation systems on lettuce yield, quality, water use efficiency, and soil C and N emissions. This study used the entropy weight coefficient evaluation method for selecting the optimal irrigation system for water saving, yield and quality improvements, and greenhouse gas emission reduction as a theoretical and practical base.

## 2. Materials and Methods

### 2.1. Experimental Site

**Figure 1:**
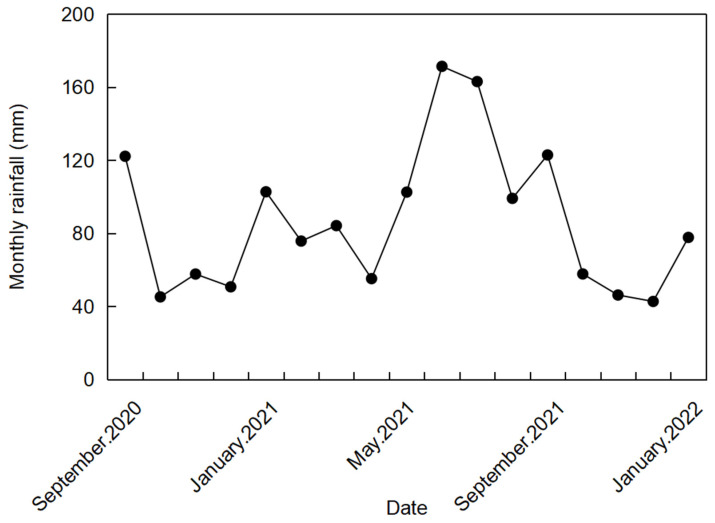
The monthly rainfall.

The experiment was conducted in the Fruit Science and Technology Field of the Modern Area (23°57′38″ N, 117°20′5″ E) of Yun Xiao County in Fujian Province, China, from September 2020 to January 2022. The altitude of Yunxiao is 320 m. The annual average temperature of the experimental field is 21.3 °C. The absolute maximum temperature is 38.1 °C, and the absolute minimum temperature is −0.2 °C. The annual precipitation in Fujian Province is 1730.6 mm. However, during the cultivation period of lettuce, an uneven distribution of rainfall often occurs. The frost-free period is 347 days. The soil type is ferrallitic soil. Soil was sampled from the top layer (0–20 cm) in the experimental site and analyzed. Soil properties are a pH of 5.9, available N content of 90.2 mg/kg, available P content of 12.2 mg/kg, available K content of 152.3 mg/kg, bulk density of 1.26 g/cm^3^, and field capacity of 0.28 g/g. The monthly rainfall is displayed in Figure 1.

### 2.2. Cropping Practices, Experimental Design, and Treatments

The lettuce variety used for this study was “*Feiqiao Lettuce No.1*”. The seeds were placed in pure water for 8 h, then encased and saved at a temperature of 5 °C for 10 h to break dormancy. During this period, the seeds were flipped once to facilitate germination. Then, the seeds were removed and set in cold conditions for about 20 h. When 2/3 of the seeds were well exposed, they were moved to the seedling tray. Seedlings with four to five leaves were transplanted in the field. The lettuce was irrigated after transplanting and when the plant reached the rosette stage.

The plot area was 32 m^2^ (4 m × 8 m). The experimental area was separated into 12 experimental blocks, and treatment used three blocks. Also, each block used the ridge planting system (height of 20 cm and width of 60 cm). The seedlings were transplanted at three ridges in each plot, with a plant-to-plant distance of 35 cm and a row-to-row interval of 30 cm. To avoid water seepage between treatments, soil ridges without any lettuce planting were placed between the FI block and the other irrigation blocks.

This experiment was conducted on the open field and employed a single-factor randomized block design with four treatments and three repetitions. The main plot was drip irrigation (DI) with three different lower irrigation limits corresponding to soil water contents at 55%, 65%, and 75% of the field capacity, named DR3, DR2, and DR1, respectively. The upper irrigation limitation of all DI was set at a soil water content of 95% of the field capacity. Furrow irrigation (FI) was employed as a control treatment, where the water was supplied to the soil ridge at 1/3 furrow height. The soil water content was measured daily using the drying method after collecting the soil samples from the top layer (8–10 cm), and irrigation was initiated when the soil moisture content reached the low limit of irrigation.

For each DI system, the water amount for DR1, DR2, or DR3 was calculated using the following equation [13]:

$$M=S\times r\times h\times Q\times \left(q^{1}-q^{2}\right)/0.95$$

where *M* represents the irrigation amount (m^3^); *S* represents the irrigated area (m^2^); r represents the bulk density (kg/m^3^); *h* represents the planned wetting layer depth (0.2 m); *Q* is the field capacity (%); *q*^1^ represents the upper irrigation limit, *q*^2^ represents the irrigation lower limit (75%, 65% or 55%), and 0.95 is the irrigation coefficient.

In local practice, farmers employ FI, and their method is to irrigate until reaching a water level that is 1/3 of the height of the furrow. In their practice, the amount of water supply for FI is similar to the calculated amount under DR2 treatment in this study.

The experiment was repeated for three seasons. The considered growth stage divisions are shown in Table 1.

Detailed information on the irrigation events, interval, quota (irrigation input at each irrigation event), and total water use implemented in this study is shown in Table 2.

Urea, calcium superphosphate, and potassium sulfate as chemical fertilizers were supplied to the experimental plots as basal and topdressing fertilizers. The chemical fertilizers used in this study had total doses of 675 kg/ha, 600 kg/ha, and 375 kg/ha for urea, calcium superphosphate, and potassium sulfate, respectively. The entire superphosphate fertilizer was supplied as the base fertilizer. However, potassium sulfate and urea were submitted as a basal fertilizer at the rate of (20%), a first topdressing at the rate of (40%) during the rosette stage, and a second topdressing at the rate of 40% in the fleshy stem expansion stage. The fertilizers were applied to a soil depth of 6 cm using a fertilization machine. Plant disease protection was applied during the whole development phase based on the local farmers’ practice, and the same practice was used for all treatments.

### 2.3. Plant Sampling and Measurements

For all three seasons, six lettuce plants were randomly selected from each block to measure the leaf area index (LAI), yield, and stem cracking rate. The plant canopy analyzer (LAI-2200, LI-COR, 4647 SUPERIOR ST. LINCOLN, NE 68504 LOUISIANA USA, Lincoln, NE, USA) was used to measure the LAI. The LAI was calculated based on the attenuation of sky scattered radiation after passing through the canopy at five zenith angle intervals (0–13°, 16–28°, 32–43°, 47–58°, 61–74°; the central zenith angles are 7°, 23°, 38°, 53°, and 68°, respectively) measured using a “fish eye” optical sensor [14]. The lettuce quality indicators, including crude fiber, vitamin, soluble sugar, and nitrate content, were measured. The crude fiber was determined using the paradigm washing method; the soluble sugar content was determined using the anthrone sulfate method; the nitrate was determined using the phenol disulfonic acid colorimetric method; and the Vc was determined using the method of 2,6-dichloro indophenol titration [15].

### 2.4. Measurements and Determination of C and N Emission Fluxes

**Figure 2:**
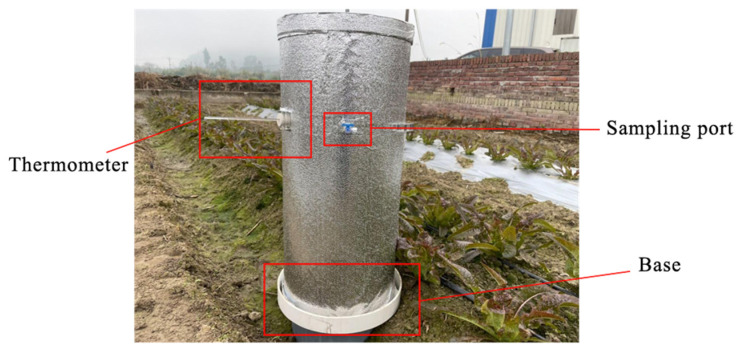
The self-made device.

In this study, a self-made gas collection apparatus was used (Figure 2). The device had a diameter of 30 cm and a height of 80 cm and consisted of a body and a base with a cover constructed from PVC material and wrapped with a silver reflective film. The device base was inserted in the soil, and the upper surface of the base contained a water tank to provide a seal. Prior to gas collection, the water tank was filled with purified water, and the cover was placed on top of the base. A thermometer and a port for collecting gas were fixed on the side of the cover. Also, a small fan powered by an external battery was positioned inside the cover to distribute the emitted gas evenly. The C and N emission flux and the accumulated C and N emission amounts were calculated according to a standard method [16]. The gas samples were collected during the third season.

### 2.5. Application of the Entropy Weight Coefficient Model

The entropy weight coefficient model was applied according to the evalution indicators and the steps are as follows [17]:

Assuming that there are *m* kinds of lettuce irrigation treatment and *n* evaluation indexes in this experiment, the matrix for evaluation can be formed by using *m* kinds of lettuce irrigation treatment corresponding to *n* different indicators (according to the design of the experiment in this study, *m* = 4, *n* = 10):

(1) $$R={\left(r_{ij}\right)}_{m\times n}$$

In the formula, *r_ij_* is the *j*th indicator value under the *i*th irrigation scheme. For a certain indicator *r_j_*, there is information entropy:

(2) $$E_{j}=-\sum_{i=1}^{m}p_{ij}\ln p_{ij},\left(j=1,2,3,\ldots n\right)$$

where, $p_{ij}=r_{ij}/\sum_{i-1}^{m}r_{ij}$.

The *j*th indicator’s entropy value can be calculated using:

(3) $$e_{j}=\frac{1}{\ln m}E_{j},\;\;\left(j=1,\;2,\;3,\ldots ,\;n\right)$$

The *j*th indicator’s objective weight can be calculated using:

(4) $$\theta_{j}=\left(1-e_{j}\right)/\sum_{i=1}^{n}(1-e_{j}),\;\;\left(j=1,\;2,\;3,\;\ldots ,\;n\right)$$

According to the calculations, it can be found that $0\leq \theta_{j}\leq 1$, $\sum_{j=1}^{n}\theta_{j}=1$.

In irrigation method decisions, the decision-makers’ experience is also important when formulating an irrigation system. Therefore, the subjective weight could be combined with the objective weight $\theta_{j}$ (*j* = 1 to *n*) of the evaluation indicators to form a new indicator weight:

(5) $$\alpha_{j}=\theta_{j}\bar{\omega_{j}}/\sum_{j=1}^{n}\theta_{j}\bar{\omega_{j}},\;\;\left(j=1,\;2,\;3,\ldots ,\;n\right)$$

If the optimum value in each list of indicators in the matrix R is ${r_{j}}^{*}$, the evaluation data can be normalized in terms of the ${r_{j}}^{*}$ value, and the ${r_{j}}^{*}$ value is commonly influenced by the nature of the indexes.

In this experiment, the lettuce yield, nitrogen fertilizer use rate, irrigation water utilization efficiency, crude fiber, soluble sugar content, and Vc content are profitability indicators, which comply with the criterion of “the greater, the better”.

The stem cracking rate, nitrate content, and carbon and nitrogen emissions are the loss indexes, which comply with the criterion of “the smaller, the better”. The standard index value *d_ij_* is formed by:

(6) $$d_{ij}=\left\{\begin{array}{l} \frac{r_{ij}}{{r_{j}}^{*}},\;\;\;\;{r_{j}}^{*}=\max\left\{r_{ij}\right\}\\ \frac{{r_{j}}^{*}}{r_{ij}},\;\;\;\;{r_{j}}^{*}=\min\left\{r_{ij}\right\} \end{array}\right.$$

The evaluation value using the entropy weight method for the irrigation schemes can be calculated as:

(7) $$\lambda_{i}=\sum_{j=1}^{n}\alpha d_{ij},\;\;i=1,\;2,\;3,\ldots ,\;m.$$

### 2.6. Statistical Analysis

Data were statistically analyzed using the SPSS (version 17.0) software. Significant differences were calculated in terms of Duncan’s multiple range test. The significant differences among indicator values of the different treatments in the same season were compared. Each value used for optimizing irrigation systems is the average of the three seasons except the C and N emissions.

## 3. Results and Analysis

### 3.1. Effect of the Different Irrigation Treatments on the Lettuce Yield

**Figure 3:**
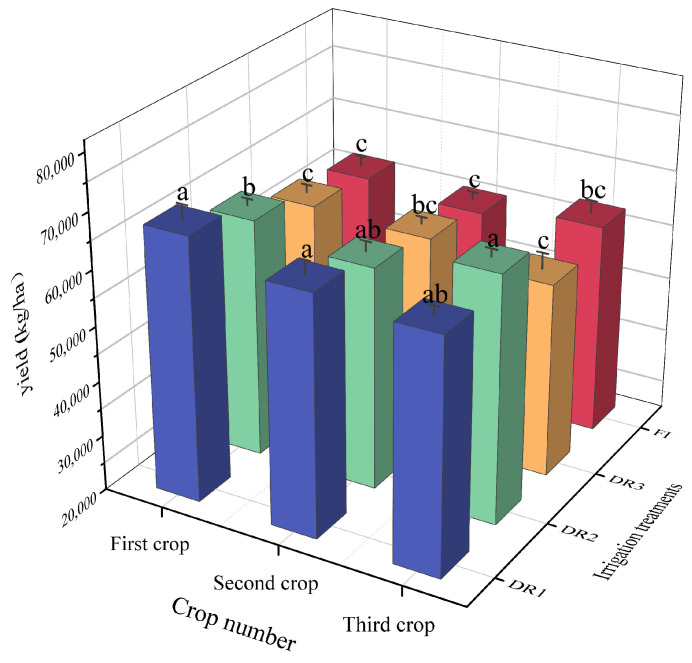
Effects of different irrigation methods on the lettuce yield. DR1, DR2, and DR3 represent lower irrigation limits at soil moisture contents of 75%, 65%, and 55% of the field capacity. FI is a furrow irrigation method using the same irrigation quota and regime as the DR2 treatment. The data are mean ± standard deviation. The significant differences among yield values of the different treatments in the same season were compared. The different letters (a, b, c) suggest significant differences at 0.05 level in terms of Duncan’s multiple range test.

The lettuces’ responses to different irrigation systems were similar between the first and second seasons, where the lettuce yield under DR1 showed a high level, reaching 67,666.5 and 63,528 kg/ha, respectively (Figure 3). Also, the yield level in the first season under DR1 was significantly higher (*p* < 0.05) than under all other treatments. Moreover, there was no significant (*p* > 0.05) difference in lettuce yield between DR3 (58,836 kg/ha) and FI (57,264 kg/ha) treatments. In the third season, the lettuce yields’ responses to different irrigation methods were slightly different from the first and second seasons, showing that the yield levels under DR1 and DR2 were higher than those in DR3 and FI. Also, there was no significant (*p* > 0.05) difference in lettuce yield between DR1 and DR2; also no noticeable difference was found between DR3 treatment and FI. From the average yield of the three seasons, DR1 resulted in the greatest average yield of 64,500 kg/ha, followed by DR2, with an average yield of 62,700 kg/ha. However, the yield of DR3 and FI were relatively low, with an average yield of 57,360 kg/ha and 57,060 kg/ha compared to the other treatments.

### 3.2. Effect of the Different Irrigation Treatments on Lettuce LAI

**Figure 4:**
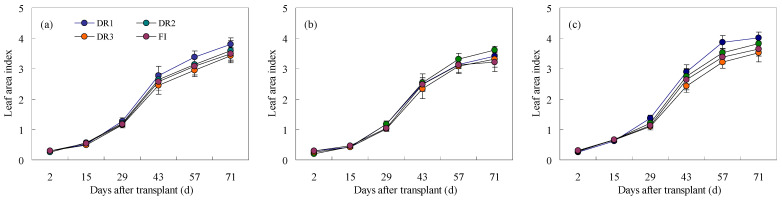
Impacts of different irrigation schemes on the LAI of lettuce. ((**a**) the first season, (**b**) the second season, and (**c**) the third season) represent the first, second, or third cultivation season of lettuce. DR1, DR2, and DR3 represent lower irrigation limits at soil moisture contents of 75%, 65%, and 55% of the field capacity. FI is a furrow irrigation method using the same irrigation quota and regime as the DR2 treatment.

The LAI presented three stages, including a slow growth stage from 2 to 29 days after transplant (DAT), a rapid growth stage from 29 to 57 DAT, and after 57 DAT (Figure 4). The differences in LAI of lettuce among different treatments began to be obvious at 43 DAT. For the first and third seasons, the highest LAI was detected under the DR1 treatment, followed by the DR2 treatment, while the lowest was in the DR3 treatment. The LAI of lettuce under FI was lower than that under DR2 treatment with the same irrigation quota. However, in the second season, the LAI under DR2 was slightly higher than under DR1, and other regularities were consistent with the first and third seasons.

**Figure 5:**
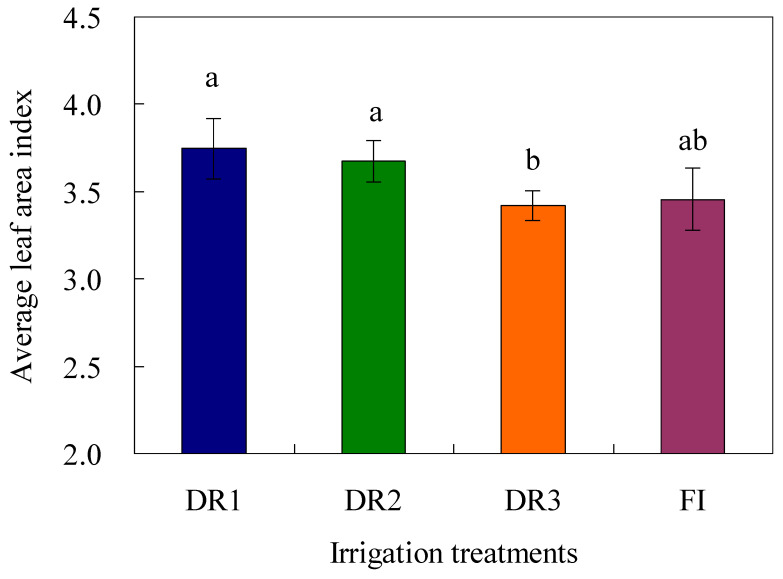
The average leaf area index of three seasons of lettuce under different irrigation methods (71 days after transplant). DR1, DR2, and DR3 represent lower irrigation limits at soil moisture contents of 75%, 65%, and 55% of the field capacity. FI is a furrow irrigation method using the same irrigation quota and regime as the DR2 treatment. The data are mean ± standard deviation. The letters, such as ‘a’ and ‘b’, suggest that there is a significant difference at the 0.05 level in terms of Duncan’s multiple range test.

Although there were clear differences in the LAI among different irrigation treatments and seasons, LAI indices under DR1 and DR2 were higher than those under DR3 and FI (Figure 5). The average LAI at 71 DAT confirmed these results. Also, it was noted that the average LAI of three seasons under DR1 treatment was at 71 DAT, reaching the highest value of 3.75, followed by DR2 (3.67). Whereas there was no significant difference in the LAI of lettuce among DR1, DR2, and FI, the LAI of lettuce under DR3 was the lowest (3.42) and was significantly (*p* < 0.05) lower in comparison to that under DR1 or DR2 treatment.

### 3.3. Effect of the Different Irrigation Treatments on the Quality of Lettuce

Among the different drip irrigation treatments, DR3 obtained higher contents of crude fiber, soluble sugar, and Vc (Table 3). This effect was evident in the third season, as the three indicators (crude fiber, soluble sugar, and Vc content) under DR3 were significantly (*p* < 0.05) greater than those under DR1 or DR2 treatments. However, while improving quality indicators, DR3 increased the nitrate content, with nitrate content reaching 412.8, 481.8, and 482.7 mg/kg for three seasons, respectively. The quality indicator values showed a downward trend as the lower irrigation limit increased.

The different Irrigation methods also have a significant impact on the quality indicators of lettuce. For the first and second seasons, drip irrigation under the same irrigation quota was beneficial to increase the contents of crude fiber, soluble sugar, and Vc in lettuce while reducing the nitrate content. There was no significant difference (*p* < 0.05) in crude fiber or soluble sugar content between DR2 and FI in the third season. However, the DR2 significantly (*p* < 0.05) increased the Vc content and decreased the nitrate content. From the comprehensive performance of lettuce quality, drip irrigation was more efficient in promoting quality improvement compared to furrow irrigation.

### 3.4. Effect of the Different Irrigation Treatments on the Cracking Rate of Lettuce

**Figure 6:**
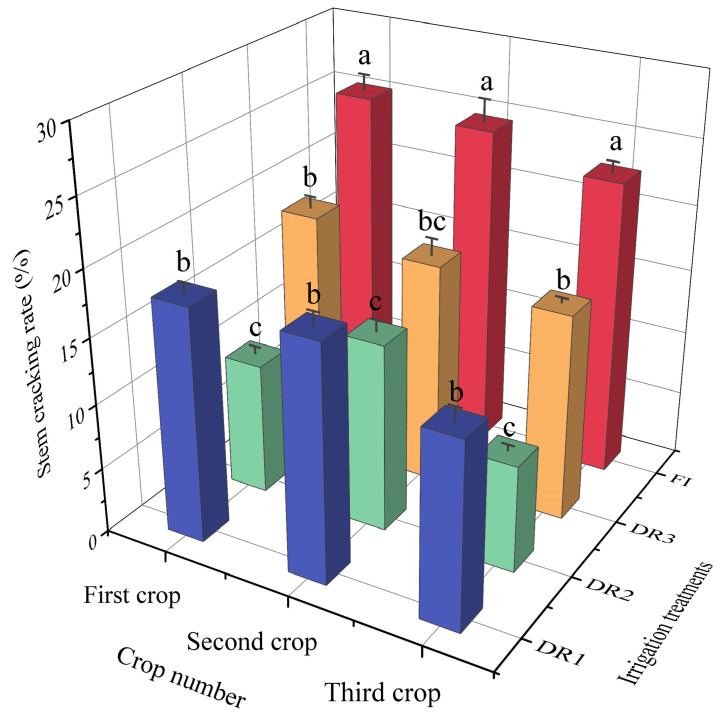
Effects of the different irrigation systems on the cracking rate of lettuce stems for the three seasons. DR1, DR2, and DR3 represent lower irrigation limits at soil moisture contents of 75%, 65%, and 55% of the field capacity. FI is a furrow irrigation method using the same irrigation quota and regime as the DR2 treatment. The data used in the figure are mean ± standard deviation. The different letters like a, b, and c suggest that there were significant differences at the 0.05 level in terms of Duncan’s multiple range test. The significant differences among the stem cracking rate values of the different treatments in the same season were compared.

There was an obvious relationship between the cracking rate of lettuce and irrigation methods. The cracking rate under FI was the highest, with the cracking rates for the three seasons reaching 25.3%, 24.6%, and 22.7%, respectively, and were significantly (*p* < 0.05) greater compared to that under the other treatments (Figure 6). Among drip irrigation treatments, DR2 showed the lowest rate of stem cracking compared to other treatments, especially in the first and third seasons. The stem cracking rates under DR2 were 10.1%, 14.4%, and 8.2%, respectively, for the three seasons, which were decreased by nearly half compared to FI. There was no significant difference in the lettuce stem cracking rate between DR1 and DR3. Drip irrigation had a significant effect on reducing the lettuce stem cracking rate, and DR2 was optimal in controlling the cracking rate.

### 3.5. Optimization of Irrigation Systems Based on Water Conservation and Emission Reduction

The evaluation indicators for lettuce irrigation systems are shown in Table 4. The yield, stem cracking rate, irrigation water efficiency, and lettuce quality indicators such as crude fiber, soluble sugar, nitrate, and Vc in the table are averaged for the three seasons. The C emissions are the sum of accumulated C emissions converted by CO_2_ and CH_4_. The N emissions are the sum of accumulated N emissions converted by N_2_O and NO.

**Figure 7:**
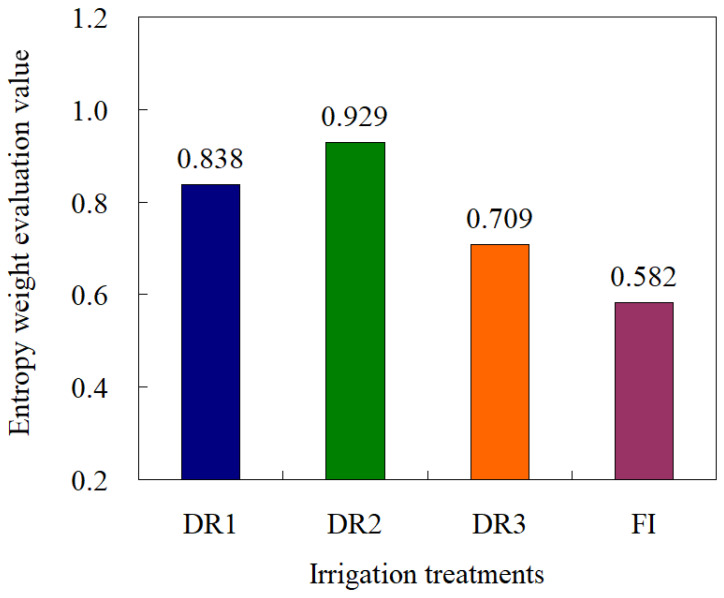
Evaluation value of entropy weight method for comprehensive benefits of water saving, yield increasing, quality improving, and emission reduction with different irrigation schemes. DR1, DR2, and DR3 represent lower irrigation limits at soil moisture contents of 75%, 65%, and 55% of the field capacity. FI is a furrow irrigation method using the same irrigation quota and regime as the DR2 treatment.

Among the different drip irrigation treatments, DR2 showed the highest evaluation value, reaching 0.93 (Figure 7), indicating that considering the four dimensions of water saving, yield increase, quality improvement, and emission reduction, the comprehensive benefits of DR2 treatment were optimal. From the perspective of indicator composition, the yield, nitrogen fertilizer utilization efficiency, and irrigation water utilization efficiency were all suboptimal, and the C and N emission reduction effect was also at the suboptimal level under DR2. However, the stem cracking rate indicator under DR2 was optimal among the four treatments. These factors led to the best evaluation result for DR2. The evaluation value under FI was lower compared to DR2, which was only 0.58. Based on the entropy weight coefficient evaluation model, DR2 can be proposed as a water-saving and emission-reduction irrigation method for lettuce fields, with an optimal low limit of drip irrigation at a soil water content of 65% of the field capacity.

## 4. Discussion

The leaves largely reflect the physiological and biochemical conditions of plants. Therefore, the LAI is an important indicator for measuring the growth characteristics of plant populations. An earlier study has shown that with sufficient water supply, the crop leaves are relatively thin, and the leaf area increases significantly [18]. On the contrary, under conditions of soil water stress, the leaves are thicker, and the leaf growth rate is slower [19]. In this study, the total water use was the highest under DR1, and the LAI under DR1 was also the greatest among the four treatments. This result was in line with the regularity summarized by previous studies [20,21].

Crop yield is formed after the solar energy is effectively transformed into chemical energy and accumulated in the crop [22]. Unreasonable soil water supply will directly affect the transformation efficiency of solar energy on crop plants, and will ultimately be reflected in the yield [23]. In this study, the average yield of DR1 was at a high level, indicating that within a certain range, irrigation frequency and irrigation amount are positively correlated with crop yield, which is consistent with previous research conclusions [22,24]. The average yields of DR3 and FI lettuce were at the middle levels. The reason may be that the DR3 can save water; however, the lettuce, due to insufficient soil moisture, suffered damage in physiological processes and, eventually, the yield formation. Conversely, under FI, soil developed saturation zones, which hinders the process of obtaining oxygen by the root system and limits the growth and development of the fleshy stem, thus reducing yield.

It is believed that the quality indicators of vegetables include taste indicators, nutritional indicators, and constraint indicators [25,26]. Due to the difficulty in quantifying taste indicators, this study selected soluble solids as the quality indicator that reflects taste. Meanwhile, the nutritional indicators, including crude fiber and Vc, the constraint indicator nitrate, were selected for the analysis of the comprehensive quality of lettuce. Herein, the average soluble sugar content of three seasons of lettuce was higher under DR3 than under other irrigation treatments, which may be due to the fact that the amount of water supply throughout the entire growth period under DR3 treatment was the lowest, which resulted in a relatively high concentration of soluble sugar in the fleshy stems of the lettuce. A previous study has shown that an increase in crude fiber is often related to low water supply, high temperature, and low fertilizer [21]. Consistently, in this study, the treatment (DR3) with a lower irrigation amount obtained the higher crude fiber content. The correlation between the average Vc content and irrigation systems in this study was similar to that between other quality indicators and irrigation methods. Our study found that the contents of crude fiber, soluble sugar, nitrate, and Vc were at the highest levels under DR3, indicating that no treatment in this study could increase the lettuce quality while reducing nitrate content. In addition, DR3 has the lowest lettuce yield, indicating that changing the irrigation method from “small quota but high frequency” to “high quota but low frequency” will decrease the lettuce yield, but improve the values of quality indicators.

The rate of stem cracking under DR3 was relatively high, which may be due to the intermittent drought accelerating the lignification of the outer epidermis of lettuce. Also, a greater irrigation quota under DR3 caused the rapid growth of the fleshy stem, and the difference in growth rate between the epidermis and fleshy stem led to the formation of stem cracking, which is consistent with the result of earlier studies [27,28]. In addition, the rate of stem cracking under DR1 was also at a high level, which may be related to local production habits.

Earlier studies on crop quality often focused on the nutritional quality or the harmful indicators such as hormones, heavy metals, and pesticide residues [29,30]. Limited attention has been paid to the marketable indicators such as the stem cracking rate. In addition, when designing the irrigation system, little attention has been paid to C and N emission indicators. Therefore, the scientific evaluation of constructing indicators of designed irrigation systems to achieve a comprehensive evaluation of this system is a major issue that needs to be addressed at present. Previous studies have found that nitrogen fertilizer input in crops may be an effective strategy to reduce carbon and nitrogen emissions, and crop yield and quality are also important indicators of crop harvest [31]. In this study, crop yield, quality, water, and nitrogen fertilizer utilization efficiency, as well as the C and N emissions as evaluation indicators, were selected. The entropy evaluation model was used to select the most suitable lower limit of irrigation for water conservation and emission reduction in lettuce fields. This study emphasizes the importance of considering C and N emissions when designing an irrigation scheme under the current environmental background of the greenhouse effect. In addition, tuberous crops commonly cause significant disturbance to soils, which greatly affects the emission of gaseous C and N from the soil, and irrigation strategies are closely related to crop root growth. Therefore, investigating suitable emission-reduced irrigation strategies for tuberous crops is more representative. Previous studies have shown that the entropy evaluation method is more accurate when there are more indicators and schemes [32]. In this experiment, four irrigation systems were implemented, which is generally at a low level compared to previous studies. One shortcoming of this study is that we only had monthly rainfall data from local meteorological stations, while the daily rainfall was not measured, which made the total rainfall at each reproductive stage unclear. The results revealed that the DR2 treatment had the optimal comprehensive benefits in consideration of water saving, yield increase, quality improvement, and emission reduction. However, the soil’s mechanical composition is crucial for soil water availability. Therefore, future research should pay attention to the soil texture and its relation to the irrigation schedule, the crop yield, and the quality of lettuce.

## 5. Conclusions

The present study revealed that drip irrigation treatments were more conducive to improving the comprehensive quality of lettuce than furrow irrigation. Among the drip irrigation treatments, DR3 obtained the higher contents of crude fiber, Vc, and soluble sugar. For all irrigation treatments, DR1 increased the crop yield significantly, and FI was found to largely increase the cracking rate of lettuce. The results of the entropy method indicated that the DR2 treatment has the optimal comprehensive benefits in consideration of water conservation, yield and quality improvement, and emission reduction. We highlighted the significance of considering soil C and N emissions when formulating an irrigation scheme, especially for tuberous crops. Further research can focus on the process and mechanism of the impact of root enlargement on soil C and N emissions under the influence of irrigation.

## Figures and Tables

**Table 1:** Detailed information on irrigation practices implemented during different growth stages.

Season Order	Date	Growth Stages	Watering Periods	Mean Temperate (°C)
First season	15 September 2020–17 September 2020	Seed germination		29.87
	18 September 2020–12 October 2020	Seedling stage		26.56
	13 October 2020–16 November 2020	Rosette stage	☆	23.82
	17 November 2020–26 December 2020	Fleshy stem expansion stage	☆	19.55
	27 December 2020–30 December 2020	Harvest stage		16.75
Second season	16 January 2021–18 January 2021	Seed germination		14.01
	19 January 2021–17 February 2021	Seedling stage		18.16
	18 February 2021–28 March 2021	Rosette stage	☆	19.43
	29 March 2021–8 May 2021	Fleshy stem expansion stage	☆	23.04
	9 May 2021–13 May 2021	Harvest stage		28.78
Third season	20 September 2021–22 September 2021	Seed germination		30.66
	23 September 2021–15 October 2021	Seedling stage		29.43
	16 October 2021–21 November 2021	Rosette stage	☆	22.59
	22 November 2021–2 January 2022	Fleshy stem expansion stage	☆	19.11
	3 January 2022–6 January 2022	Harvest stage		16.25

Note: ☆ denotes that plants were grown under different irrigation treatments during a specific growth stage.

**Table 2:** The irrigation times, interval, quota, and total water used during different growth stages.

Season Order	Treatments	The Stage from Rosette to Fleshy Stem Expansion				Total Water Use (mm)
		Irrigation Times	Irrigation Interval (d)	Irrigation Quota (mm)	Irrigation Amount (mm)	
First season	DR1	8	8.7	15.8	126.5	299.1
	DR2	5	14.0	23.7	118.6	291.2
	DR3	3	23.5	31.6	94.9	267.5
	FI	5	14.0	23.7	118.6	291.2
Second season	DR1	7	11.1	15.8	110.6	275.7
	DR2	4	19.5	23.7	94.8	259.9
	DR3	2	39.0	31.6	63.2	228.3
	FI	4	19.5	23.7	94.8	259.9
Third season	DR1	8	9.5	15.8	126.5	284.1
	DR2	5	15.2	23.7	118.6	276.2
	DR3	3	25.3	31.6	94.9	252.5
	FI	5	15.2	23.7	118.6	276.2

Note: DR1, DR2, and DR3 were drip irrigation treatments, and FI was furrow irrigation.

**Table 3:** Effect of the different irrigation methods on the quality of lettuce for the three seasons.

Season Order	Treatment Applied	Crude Fiber (%)	Soluble Sugar (%)	Nitrate (mg/kg)	Vitamin c (mg/kg)
First season	DR1	2.67 ± 0.21 b	4.39 ± 0.12 c	334.8 ± 9.5 c	65.2 ± 5.6 b
	DR2	3.22 ± 0.25 a	4.92 ± 0.08 a	362.8 ± 13.2 b	83.6 ± 6.7 a
	DR3	3.56 ± 0.32 a	5.08 ± 0.15 a	412.8 ± 21.8 a	84.8 ± 6.8 a
	FI	3.11 ± 0.13 a	4.66 ± 0.09 b	388.4 ± 24.2 ab	64.1 ± 5.4 b
Second season	DR1	2.78 ± 0.25 c	4.60 ± 0.15 b	419.1 ± 21.6 b	75.1 ± 4.2 b
	DR2	3.81 ± 0.32 ab	5.35 ± 0.35 a	422.7 ± 19.1 b	93.7 ± 7.1 a
	DR3	3.92 ± 0.11 a	5.02 ± 0.18 a	481.8 ± 29.7 a	87.6 ± 4.2 a
	FI	3.71 ± 0.06 b	4.45 ± 0.19 b	440.7 ± 31.5 ab	77.1 ± 2.9 b
Third season	DR1	3.22 ± 0.15 c	4.63 ± 0.15 b	392.6 ± 19.2 c	73.3 ± 3.9 c
	DR2	3.62 ± 0.08 b	4.61 ± 0.19 b	412.3 ± 25.6 bc	85.7 ± 2.5 b
	DR3	3.86 ± 0.11 a	5.26 ± 0.36 a	482.7 ± 18.3 a	92.3 ± 1.5 a
	FI	3.77 ± 0.15 ab	4.72 ± 0.29 ab	434.6 ± 15.8 b	67.5 ± 4.8 c

Note: DR1, DR2, and DR3 represent lower irrigation limits at soil moisture contents of 75%, 65%, and 55% of the field capacity. FI is a furrow irrigation method using the same irrigation quota and regime as the DR2 treatment. The different letters (a, b, c) suggest significant differences at 0.05 level in terms of Duncan’s multiple range test. The significant differences among quality indicator values of the different treatments in the same season were compared.

**Table 4:** The evaluation indicators for lettuce grown under different irrigation systems.

Treatment	Yield (kg/ha)	Stem Cracking Rate (%)	Nitrogen Use Efficiency (%)	Irrigation Water Use Efficiency (kg/m^3^)	Crude Fiber (%)	Soluble Sugar (%)	Nitrate (mg/kg)	Vc (mg/kg)	C Emission (g C/m^2^)	N Emission (kg/ha)
DR1	64,500	16.7	41.6	22.52	2.89	4.54	382.16	71.21	344.7	2.10
DR2	62,700	10.9	41.5	22.73	3.55	4.96	399.26	87.68	459.6	2.41
DR3	57,360	17.2	37.3	22.98	3.78	5.12	459.12	88.23	483.4	2.99
FI	57,060	24.2	34.7	20.68	3.53	4.61	421.23	69.58	382.7	3.95

Note: DR1, DR2, and DR3 represent lower irrigation limits at soil moisture contents of 75%, 65%, and 55% of the field capacity. FI is a furrow irrigation method using the same irrigation quota and regime as the DR2 treatment.

## Data Availability

The data presented in this study are available on request from the corresponding author. The data are not publicly available due to the safety of the project. The relevant data are confidential and cannot be published in this article.
